# Nephroprotective Effect of the Virgin Olive Oil Polyphenol Hydroxytyrosol in Type 1-like Experimental Diabetes Mellitus: Relationships with Its Antioxidant Effect

**DOI:** 10.3390/antiox10111783

**Published:** 2021-11-08

**Authors:** María Dolores Rodríguez-Pérez, Juan Antonio López-Villodres, María Monsalud Arrebola, Esther Martín-Aurioles, África Fernández-Prior, Alejandra Bermúdez-Oria, María Carmen Ríos, José Pedro De La Cruz, José Antonio González-Correa

**Affiliations:** 1 Department of Pharmacology, Faculty of Medicine, Biomedical Research Institute (IBIMA), University of Malaga, 29010 Málaga, Spain; loladoct@uma.es (M.D.R.-P.); correa@uma.es (J.A.G.-C.); 2 Area of Human Histology, Faculty of Medicine, University of Malaga, 29010 Málaga, Spain; jantoniolv@uma.es (J.A.L.-V.); mcrios@uma.es (M.C.R.); 3 Clinical Laboratory, Clinical Management Unit, Hospital Axarquía, AGSEMA, 29740 Málaga, Spain; mariam.arrebola.sspa@juntadeandalucia.es; 4 Clinical Management Unit La Roca, Distrito Sanitario, AGSEMA, 29740 Málaga, Spain; esther.uma@hotmail.com; 5 Department of Food Phytochemistry, Instituto de la Grasa (Spanish National Research Council, CSIC), 41013 Seville, Spain; mafprior@ig.csic.es (Á.F.-P.); aleberori@ig.csic.es (A.B.-O.)

**Keywords:** hydroxytyrosol, diabetes, nephropathy, virgin olive oil

## Abstract

The aim of this study was to determine whether hydroxytyrosol administration prevented kidney damage in an experimental model of type 1 diabetes mellitus in rats. Hydroxytyrosol was administered to streptozotocin-diabetic rats: 1 and 5 mg/kg/day p.o. for two months. After hydroxytyrosol administration, proteinuria was significantly reduced (67–73%), calculated creatinine clearance was significantly increased (26–38%), and the glomerular volume and glomerulosclerosis index were decreased (20–30%). Hydroxytyrosol reduced oxidative and nitrosative stress variables and thromboxane metabolite production. Statistical correlations were found between biochemical and kidney function variables. Oral administration of 1 and 5 mg/kg/day of hydroxytyrosol produced an antioxidant and nephroprotective effect in an experimental model of type 1-like diabetes mellitus. The nephroprotective effect was significantly associated with the systemic and renal antioxidant action of hydroxytyrosol, which also influenced eicosanoid production.

## 1. Introduction

Diabetes mellitus is the most prevalent endocrine disease globally, affecting 6.1% of the world population [1]. Sustained uncontrolled hyperglycemia over time leads to vascular complications, most notably macroangiopathy and diabetic microangiopathy, including diabetic retinopathy, neuropathy, and nephropathy.

Diabetic nephropathy is the most serious microangiopathic complication that can occur in the evolution of a patient with diabetes mellitus. Individuals with diabetes have a nearly twofold greater likelihood of developing chronic kidney disease than those without diabetes. It is estimated that approximately 180 million patients with diabetes mellitus worldwide have some form of kidney injury [2]. Although diabetic nephropathy remains silent throughout life in some of these patients [3], when this complication is not well controlled, most patients will eventually need dialysis and, possibly, kidney transplantation. Moreover, in the United States, mortality among patients with diabetic nephropathy is four times higher than in patients with nondiabetic chronic kidney disease [2]. Therefore, in addition to maintaining proper control of hyperglycemia, prophylactic compounds are also being studied.

Persistently high glucose concentrations alter renal cell morphology, mainly in the glomeruli [4]. At the biochemical level, several mechanisms are involved in diabetic nephropathy, which basically coincide with those affecting diabetic vasculopathy [4] including oxidative stress, nitrosative stress, inflammation, induction of growth mediators, and activation of the renin–angiotensin system [5]. In the pathophysiology of diabetic vasculopathy and diabetic nephropathy, oxidative stress caused by persistent hyperglycemia is one of the first biochemical events that initiate these lesions and enhance other pathways of cellular damage [6]. Accordingly, the potential use of antioxidant compounds in the prevention of diabetic nephropathy has been postulated [7].

One of the most important sources of antioxidant compounds is the Mediterranean diet, which has been proposed as beneficial in the prevention of diabetic nephropathy [8]. Extra virgin olive oil (EVOO) is the main source of fats and antioxidant compounds in the Mediterranean diet [9]. The renal protective effect of EVOO has been studied in experimental models of nephropathy, in which its antioxidant action plays an important role [10,11]. The polyphenolic compounds in EVOO are the main components responsible for its antioxidant action, with the most important of these being hydroxytyrosol [12].

In studies carried out in an experimental model of type 1 diabetes, hydroxytyrosol has been shown to slow retinal damage [13] and reduce certain cardiovascular biomarkers [14]. The main objective of this study was, therefore, to assess whether the administration of hydroxytyrosol prevents kidney damage in an experimental model of type 1 diabetes mellitus in rats. As secondary objectives, we considered whether these modifications were associated with changes in renal and serum parameters of certain biomarkers of oxidative and nitrosative stress.

## 2. Material and Methods

### 2.1. Material

Thiobarbituric acid reactive substances, total antioxidant capacity colorimetric kits, 3-nitrotyrosine, 8-iso-prostaglandin F_2α_ (8-isoprostane), and 8-hydroxy-2-deoxyguanosine enzyme immunoassay kits were obtained from Cell Biolabs Inc. (Bionova Científica S.L., Madrid, Spain). Glutathione peroxidase and total glutathione colorimetric kits were obtained from Abcam (Cambridge, UK) and 11-dehydro-tromboxane B2 and 6-keto-prostaglandin F1α enzyme immunoassay kits from Cayman Chemical Co., (Ann Arbor, MI, USA). Oxidized low-density lipoprotein immunoassay kits were obtained from Abyntec Biopharma S.L., Bizkaia, Spain. All other reagents were from Sigma Chemical Corp. (St. Louis, MO, USA).

Hydroxytyrosol was isolated by hydrothermal treatment of the liquid phase obtained from alperujo (a by-product of the two-phase olive oil separation system) at 160 °C for 60 min [15]. The liquid was extracted by two-step chromatography fractionation. The final yield reached 99.6% purity relative to dry matter, according to the process described by Fernández-Bolaños et al. [16]. The phenols were quantified using a Hewlett-Packard 1100 liquid chromatography system with an ultraviolet/visible detector. A Mediterranea Sea C18 analytical column (250 × 4.6 mm i.d.; particle size = 5 µm) (Teknokroma, Barcelona, Spain) was used at room temperature. The system was equipped with Rheodyne injection valves (20 μL loop). The mobile phases were 0.01% trichloroacetic acid in water and acetonitrile, with the following gradient during a total run time of 55 min: 95% initially, 75% at 30 min, 50% at 45 min, 0% at 47 min, 75% at 50 min, and 95% at 52 min until the run was complete. Quantification was carried out by peak integration at 280 nm wavelength with reference to calibrations obtained with external standards.

### 2.2. Study Design

The animals were 2-month-old adult male Wistar rats (body weight 200–250 g). All rats were used in accordance with current Spanish legislation for animal care, use and housing (EDL 2013/80847, BOE-A-2013-6271). The recommendations of the Guide for the Care and Use of Laboratory Animals (NIH publication No. 86-23, revised 1985) were followed, as well as the Spanish Law on the Protection of Animals, where applicable. The study protocol was approved by the University of Malaga Ethics Committee for the Use of Animals (Ref. CEUMA31-2018-A) and the Consejería de Agricultura, Ganadería, Pesca y Desarrollo Sostenible, Junta de Andalucía (Department of Agriculture, Livestock, Fisheries, and Sustainable Development of the Regional Government of Andalusia) (Ref. 9/07/2019/124). The study is reported in accordance with ARRIVE guidelines (https://arriveguidelines.org, (accessed on 17 March 2021)).

The animals (*n* = 40, 10 rats per group) were allocated into four groups (a single animal in each cage): (1) control nondiabetic rats treated with saline (NDR), (2) control diabetic rats (DR) treated with saline, (3) DR rats treated with 1 mg/kg/day p.o. hydroxytyrosol (HT-1), and (4) DR treated with 5 mg/kg/day p.o. hydroxytyrosol (HT-5). These doses were chosen based on previous results with hydroxytyrosol to analyze certain biomarkers in the present study [13,14]. Hydroxytyrosol was administered in the drinking water once daily for 7 days before diabetes was induced and continued daily until the end of the diabetic period (2 months).

Experimental diabetes was induced with a single intraperitoneal injection of streptozotocin (50 mg/kg). Blood glucose concentration was measured by placing a FreeStyle glucometer (Abbot Laboratories S.A., Madrid, Spain) in contact with blood from the saphenous vein. Animals were considered to have diabetes when blood glucose was higher than 200 mg/dL for two consecutive days. Rats in the nondiabetic control group received a single intraperitoneal injection of isotonic saline solution, and blood glucose was measured in the same way as in the diabetic animals.

During the follow-up period, the diabetic animals were treated with 4 IU/day s.c. of a soluble long-acting basal insulin analog (Levemir^®^, Novo Nordisk A/S, Bagsværd, Denmark) to reduce mortality due to the high levels of blood glucose. Control animals received the same volume of isotonic saline solution s.c. The daily amount of food and water intake was recorded throughout the follow-up period. The animals were weighed weekly to adjust the dose of hydroxytyrosol in mg/kg. At the end of the follow up, all rats were anesthetized with pentobarbital sodium (40 mg/kg i.p.) and then decapitated with a guillotine. No rats were euthanized whilst conducting the study.

### 2.3. Analytical Techniques

All techniques were run in a single-blind manner, i.e., the individuals who performed the assays were unaware of the origin and nature of the samples.

#### 2.3.1. Samples

The following samples were extracted from each animal:

− Whole blood, collected in tubes without anticoagulants and with coagulation activator gel. The samples were centrifuged at 3500× *g* for 10 min and the supernatant was separated and frozen in aliquots at −80 °C until determination of the corresponding variables.
− Kidneys: Both kidneys were perfused with isotonic saline by cannulation of the renal artery to eliminate the blood in the renal vessels. Subsequently, they were weighed, the cortex was separated from the medullary tissue, and the left renal cortex was homogenized in 50 mM phosphate-buffered saline, pH 7.0 (1/15 *w*/*v*), centrifuging the resulting sample at 13,000× *g* for 15 min at 4 °C, separating the supernatant, and freezing the aliquots at −80 °C until determination of the corresponding variables. The right kidney was used for histological analysis.
− Urine. Rats were individually placed in modular metabolic cages (Tecniplast S.p.A., Buguggiate, Italy) and 24 h urine was collected. Total diuresis was measured, and the samples were centrifuged at 3500× *g* for 10 min at 4 °C and frozen at −80 °C in aliquots until the corresponding analytical determinations were made.

#### 2.3.2. Serum and Urine Biochemistry

All biochemical parameters were analyzed using the Atellica^®^ CH autoanalyzer from Siemens Healthineers (Erlangen, Germany). Glucose concentration was determined by an enzymatic method using hexokinase and glucose-6-phosphate dehydrogenase. Creatinine determination was based on a reaction with picric acid in an alkaline medium. Total proteins were measured using cupric sulfate in alkaline solution (biuret method). Albumin was measured with bromocresol purple. Urine pH was determined with commercial Sysmex^®^ test strips (Sysmex España, S.L., Barcelona, Spain), using the UC-3500 autoanalyzer (Sysmex España, S.L., Barcelona, Spain), and pH was measured by reflectance photometry.

Creatinine clearance was calculated using the following formula [17]:

$$\frac{\mathrm{Urine}\;\mathrm{creatinine}\;\left(\mathrm{mg}/\mathrm{dL}\right)\times \;\mathrm{urine}\;\mathrm{volume}\;\left(\mathrm{mL}\right)}{\mathrm{Serum}\;\mathrm{creatinine}\;\left(\mathrm{mg}/\mathrm{dL}\right)}\;\times \;\frac{1000}{\mathrm{Body}\;\mathrm{weight}\;\left(g\right)}\;\times \;\frac{1}{1440}$$

Values are expressed in mL/min/kg body weight.

#### 2.3.3. Oxidative and Nitrosative Stress

Malondialdehyde is the main product of reaction with thiobarbituric acid (TBARS) and was used as an index of serum and tissue lipid peroxide concentration. Serum oxidized low-density lipoprotein was measured as an index of oxidative status caused by free radicals. Urinary 8-isoprostane was determined as a global index of oxidative stress [18]. Serum and kidney glutathione concentration, glutathione peroxidase activity, and total antioxidant capacity were determined as a global index of antioxidant defense. Serum and kidney 8-hydroxy-2-deoxyguanosine concentrations were determined as an index of oxidative stress/DNA damage. Serum and kidney 3-nitrotyrosine concentrations were determined as an index of peroxynitrite formation.

All these determinations were carried out according to the manufacturers’ protocols.

#### 2.3.4. Eicosanoids

Urinary 11-dehydro-thromboxane B_2_ and 6-keto-prostaglandin F_1α_ concentrations were measured as an index of the global production, including that from the kidney, of thromboxane and prostacyclin. These determinations were made according to the manufacturer’s protocols.

#### 2.3.5. Morphological Procedure

The right kidney from each rat was fixed in 10% neutral buffered formalin and processed for 5 μm paraffin sections for standard hematoxylin and eosin and periodic acid–Schiff (PAS) reaction staining methods.

Morphometric measurements of stained sections were performed in an image analysis system: histological images were taken with a virtual slide microscope (Olympus BX-UCB, with VS-ASW FL software, Hamburg, Germany). Glomerular images were then obtained with the QuPach-0.2.3 program and morphometric parameters with the FIJI ImageJ program (https://imagej.nih.gov/ij/download.html, (accessed on 5 June 2021)).

To determine glomerular volume (GV), the glomerular area (GA) of 50 glomeruli was measured as follows [19]:

GV = (GA)^3/2^ × β/d

where β is a dimensionless shape coefficient (β = 1.0 for perfect spheres), and d is a size distribution coefficient used to adjust for variations in glomerular size.

The rate of glomerulosclerosis was calculated from the PAS-stained slices in 50 glomeruli per slice. The area of each glomerulus was quantified, and the PAS-positive area was calculated in these glomeruli using the image analysis program, as follows:

GMS = [PAS(+)A (µm^2^)/GA (µm^2^)] × 100

where GMS is the percentage of glomerular area with PAS(+) material, PAS(+)A is the area occupied with PAS(+) material in a glomerulus, and GA is the area of this glomerulus.

### 2.4. Statistical Analysis

The data in the text, tables, and figures are expressed as the mean ± standard error of the mean (SEM) of 10 animals. All statistical analyses were done with the Statistical Package for Social Sciences v. 25.0 (SPSS Co., Chicago, IL, USA). One-way analysis of variance followed by Bonferroni transformation and unpaired Student’s *t*-tests were used. To establish a possible relationship between aortic morphological data and other biochemical variables, Pearson correlation coefficients were calculated. In all cases, statistical significance was assumed at a value of *p* < 0.05.

## 3. Results

**Figure 1:**
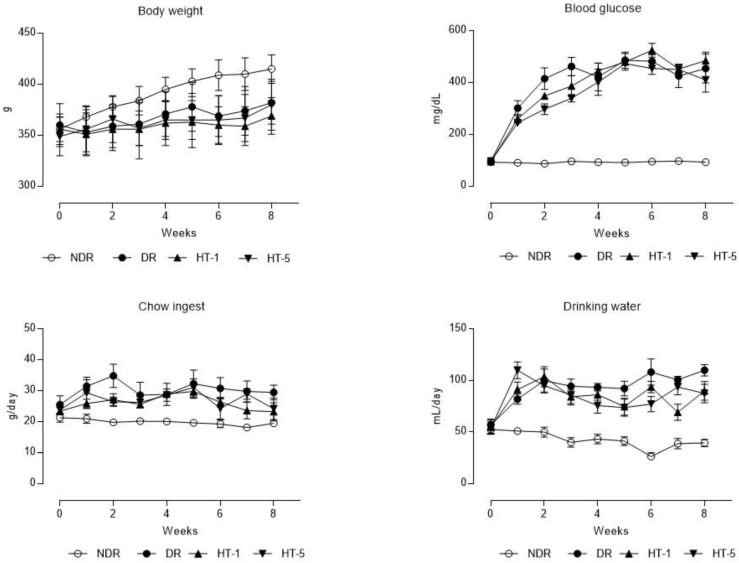
Time-course curves of the evolution of body weight, blood glucose, mean daily chow ingestion, and drinking water (mean ± standard error of the mean) in control nondiabetic rats (NDR), control diabetic rats (DR), and DR treated with hydroxytyrosol 1 mg/kg/day p.o. (HT-1) or 5 mg/kg/day p.o. (HT-5). N = 10 rats per group.

Diabetic animals showed a lower body weight evolution than the nondiabetic ones, the administration of hydroxytyrosol did not modify this variable significantly (Figure 1). On the other hand, all diabetic animals presented blood glucose levels much higher than nondiabetic animals; only in the group treated with the dose of 5 mg/kg/day p.o., blood glucose levels were reduced in the first month of treatment, but no significant differences were observed at the end of the study period (Figure 1). Finally, diabetic animals, controls, and those treated with hydroxytyrosol, ingested a greater quantity of feed and water daily (Figure 1). The kidneys of the diabetic control animals showed a higher relative weight (100 × kidney weight/body weight) than the healthy controls (0.6 ± 0.04 vs. 0.8 ± 0.04, *p* < 0.05), hydroxytyrosol did not modify this parameter even with 1 mg/kg/day p.o. (0.7 ± 0.03) or with 5 mg/kg/day p.o. (0.7 ± 0.02).

The serum biochemical profile (Table 1) showed a significant increase in glucose levels in all groups of diabetic animals. Creatinine levels doubled in the diabetic animals and then decreased after hydroxytyrosol administration (42.8% reduction with 1 mg/kg/day p.o. and 28.5% with 5 mg/kg/day p.o.). The rest of the variables showed no significant changes.

**Figure 2:**
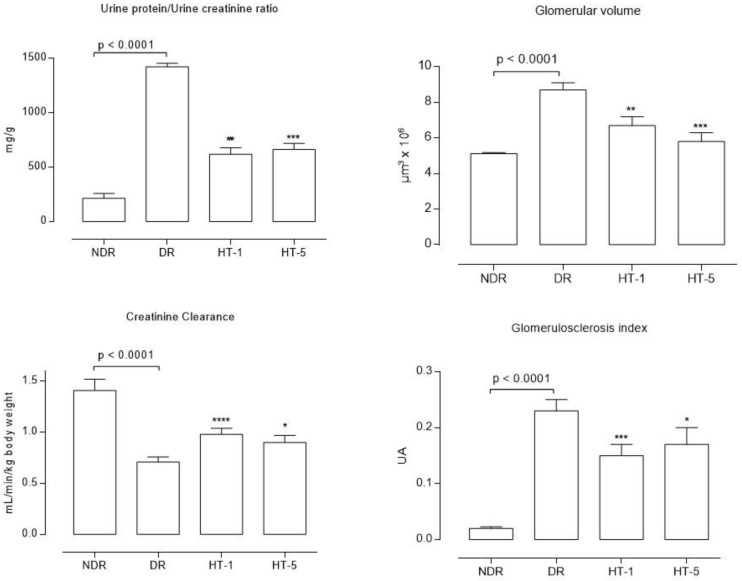
Mean values (mean ± standard error of the mean) of the proteinuria/urine creatinine ratio, calculated creatinine clearance, glomerular volume, and glomerulosclerosis index after eight weeks of follow up in control nondiabetic rats (NDR), control diabetic rats (DR), and DR treated with hydroxytyrosol 1 mg/kg/day p.o. (HT-1) or 5 mg/kg/day p.o. (HT-5). N = 10 rats per group. * *p* = 0.04, ** *p* = 0.001, *** *p* = 0.004, **** *p* < 0.0001, with respect to DR.

Urine samples (Table 1) showed a significant level of glucosuria, which was unchanged after hydroxytyrosol treatment. Urinary creatinine levels were significantly reduced in diabetic control animals, increasing after hydroxytyrosol treatment. Proteinuria (Table 1 and Figure 2) was significantly higher in diabetic animals (increase factor = 5.9), decreasing after the administration of 1 mg/kg/day p.o. (67.8%) and 5 mg/kg/day p.o. (72.9%) of hydroxytyrosol. Calculated creatinine clearance (Figure 2) was significantly lower in diabetic control animals (reduction of 49.6% compared to healthy animals), increasing after treatment with 1 mg/kg/day p.o. (38.0% increase compared to diabetic controls) and 5 mg/kg/day p.o. (26.7% increase compared to diabetic controls) of hydroxytyrosol.

**Figure 3:**
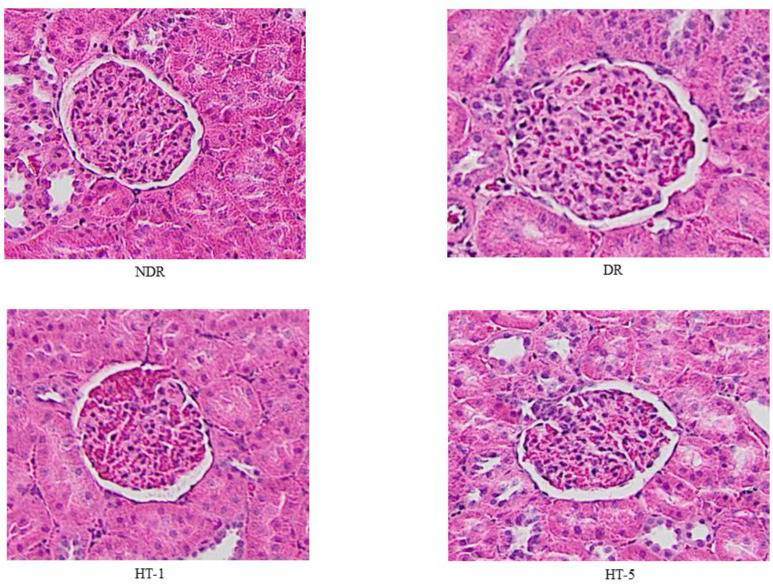
Representative examples of glomerular images from control nondiabetic rats (NDR), control diabetic rats (DR), and DR treated with hydroxytyrosol 1 mg/kg/day p.o. (HT-1) or 5 mg/kg/day p.o. (HT-5). Hematoxylin–eosin (×10).

**Figure 4:**
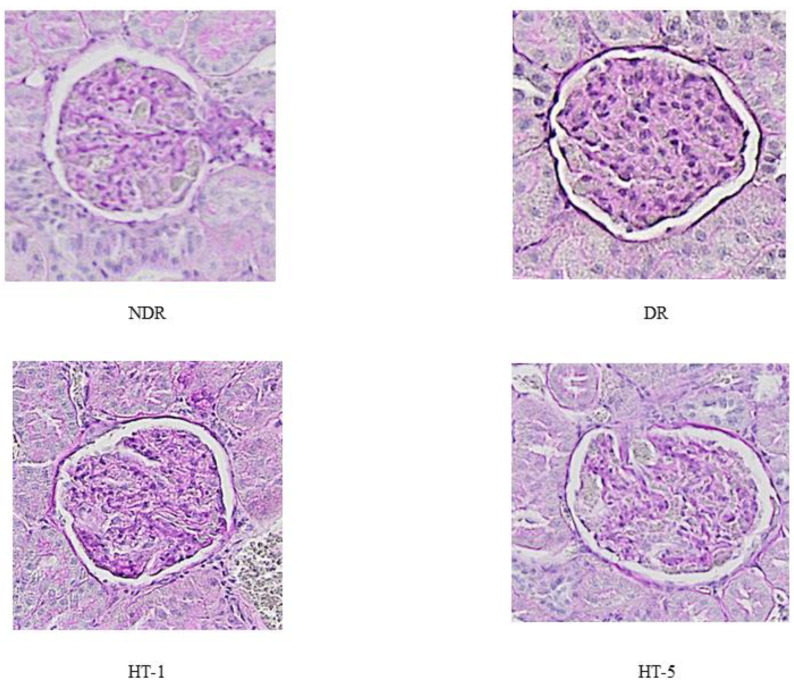
Representative examples of glomerular images from control nondiabetic rats (NDR), control diabetic rats (DR), and DR treated with hydroxytyrosol 1 mg/kg/day p.o. (HT-1) or 5 mg/kg/day p.o. (HT-5). PAS (×10).

Glomerular volume (Figure 2 and Figure 3) increased significantly in diabetic control animals (69.1% compared to healthy controls), decreasing after the administration of 1 mg/kg/day p.o. (22.1% decrease) and 5 mg/kg/day p.o. (32.8% decrease) of hydroxytyrosol. Similarly, the glomerulosclerosis index (Figure 2 and Figure 4) increased in the diabetic control animals (21.2% higher than in healthy control animals), decreasing after the administration of 1 mg/kg/day p.o. (13.9% decrease) and 5 mg/kg/day p.o. (18.1% decrease) of hydroxytyrosol.

All oxidative stress variables both in serum and kidney tissue (Table 2) were altered in the diabetic control animals. The markers of oxidative damage were significantly increased, and those of antioxidant defense were decreased. Likewise, the concentration of 3-nitrotyrosine (nitrosative stress) was also increased. Hydroxytyrosol administration reduced this imbalance significantly, except for the serum concentration of oxidized low-density lipoprotein with 1 mg/kg/day (Table 2) and the decrease in antioxidant defense quantified in the renal tissue of the diabetic controls, which did not change with either of the doses used. Finally, the concentration of 3-nitrotyrosine, which was elevated in the diabetic controls, decreased significantly, both in serum and in kidney tissue, with both doses of hydroxytyrosol.

The production of eicosanoids and 8-isoprostane was also altered in the diabetic control animals (Table 1), showing an increase in 11-dH-TxB_2_ and urinary 8-isoprostane and a decrease in 6-keto-PGF_1α_. The administration of hydroxytyrosol reduced the production of 11-dH-TxB_2_ and 8-isoprostane. However, only the dose of 5 mg/kg/day p.o. reduced the decrease in 6-keto-PGF_1α_ quantified in the diabetic control animals.

Table 3 shows the correlations between the main variables and three fundamental parameters of renal function: glomerular volume, creatinine clearance, and proteinuria (protein/creatinine ratio in urine).

## 4. Discussion

The results of this study show that hydroxytyrosol administered orally for 8 weeks to rats with experimental type 1-like diabetes mellitus reduced the main variables related to kidney damage caused by persistent hyperglycemia over time. An association between the protective effect and antioxidant action of hydroxytyrosol was also demonstrated both in kidney tissue and in serum.

An experimental model of alloxan-induced diabetes has shown that the administration of 20 mg/kg/day i.p. of a purified hydroxytyrosol extract for 8 weeks, produces an antioxidant and nephroprotective effect (observed qualitatively) [20]. Likewise, a study in an experimental model of type 2 diabetes [21] shows that the oral administration of 10 mg/kg/day of HT for 8 weeks produces an antioxidant effect and a histological improvement of the kidneys, albeit qualitatively. Hydroxytyrosol administration has also been shown to reduce the nephrotoxicity produced by drugs such as gentamicin (hydroxytyrosol: 2 mg/kg/day p.o.) [22] or cyclosporine (hydroxytyrosol: 20 × 2 mg/kg/day i.p.) [23]. Because type 1 diabetes mellitus is a life-long condition, we believe that oral administration of a potentially nephroprotective compound could improve medication adherence, considering the extended period of time for which it would need to be taken.

It is possible that the nephroprotective effect of hydroxytyrosol could be due to a normalization of glucose levels, which is fundamental in the prevention of diabetic microangiopathy. However, in this study, this reduction in blood glucose levels was not observed. The effects observed for hydroxytyrosol must, therefore, be due to a direct action on the mechanisms of kidney damage in diabetes mellitus. Some studies show an antidiabetic effect of hydroxytyrosol [24], though these studies involve models of type 2 diabetes in which the problem is the lack of insulin sensitivity in the tissues, which did not occur in the model used in our study because streptozotocin nullifies the ability of the pancreas to produce insulin. The insulin administered in our study was not intended to normalize blood glucose levels, but rather to prevent the death of the animals while maintaining a hyperglycemic state. In addition, the polydipsia and polyphagia that characterize type 1 diabetes mellitus were not modified by hydroxytyrosol administration.

Diabetic rats present alterations in the main analytical variables associated with nephropathy (proteinuria and decreased creatinine clearance), as well as glomerular morphological alterations (increased glomerular volume and glomerulosclerosis). The administration of hydroxytyrosol reduced proteinuria, glomerulosclerosis index, and glomerular volume and increased creatinine clearance. Proteinuria reduction is associated with a slower progression of nephropathy [4], for which intensive control of hyperglycemia is essential [25]. However, this glycemic control is not always achieved. Accordingly, there is a need to find drugs or compounds that facilitate this protective effect. In this regard, our findings show that hydroxytyrosol administered preventively had a nephroprotective effect in the experimental model used in this study.

In the first stages of diabetic microangiopathy, high glucose levels sustained over time originate a series of cellular biochemical changes that produce modifications in cellular functionality. One of these biochemical changes is the induction of cellular oxidative stress [26]. Oxidative stress is recognized as one of the main explanations of these cellular alterations, modifying other biochemical pathways and producing, for example, endothelial dysfunction leading to the abnormal functioning of blood vessels, both in large arteries and microvasculature [27]. In the kidney, as in other organs, diabetes mellitus has been shown to increase oxidative and nitrosative stress, causing an increase in cellular inflammatory pathways and the expression of growth factors [17,28]. All this causes defective glomerular function and morphological changes associated with an increase in cell proliferation and glomerular sclerosis [17,28]. In this study, a clear increase in general oxidative and nitrosative stress was demonstrated, both in the peripheral circulation and in the kidney tissue of the diabetic animals (Table 2). This same biochemical profile was found in a similar experimental model in brain tissue and large peripheral arteries [13,14,29].

This increase in oxidative stress (greater oxidative damage and lower antioxidant defense) and nitrosative stress (increase in the concentration of 3-nitrotyrosine) is reduced by the early administration of hydroxytyrosol, both at the peripheral level and in renal tissue. This behavior has been demonstrated in a similar experimental model in brain tissue [29]. The antioxidant effect of hydroxytyrosol, both in oxidative and nitrosative stress, has been widely demonstrated in various tissues and in different experimental models, with this effect being attributed to the benefits of hydroxytyrosol as an organ-protective compound [30]. In addition, a finding that supports the association between these factors and endothelial dysfunction that could be involved in glomerular damage is the imbalance produced by diabetes in the formation of eicosanoids (thromboxane and prostacyclin), fundamental elements for the correct maintenance of blood flow in different organs [31]. Diabetic animals showed an increase in the concentration of thromboxane (vasoconstrictor) and a decrease in the concentration of prostacyclin (vasodilator), with an increase in the amounts of prostaglandins generated by free radicals (8-isoprostanes). Hydroxytyrosol administration reduced urinary excretion of thromboxane and 8-isoprostane and slowed the decline in prostacyclin production (Table 1). The effect of hydroxytyrosol on the thromboxane/prostacyclin balance has been demonstrated in a similar experimental model and in human blood samples [13,14,32]. This effect is mainly due to an inhibition of platelet cyclooxygenase and a reduction in the biodegradation exerted by free radicals on prostacyclin [14,32]. Regarding the reduction of 8-isoprostane, this could be explained by its antioxidant effect, which would decrease free-radical-induced prostaglandin formation.

We can, therefore, hypothesize that hydroxytyrosol exerts a nephroprotective effect, which is at least partly due to its general and local antioxidant action in the kidney. This hypothesis is based on the significant association found between the different oxidative and nitrosative stress variables and three renal variables representative of kidney damage (proteinuria, creatinine clearance, and glomerular volume) (Table 3). These correlations demonstrate that the modifications made in oxidative and nitrosative stress influence kidney function in this experimental diabetes model regarding the damage caused by diabetes and its prevention after oral hydroxytyrosol administration.

Other compounds with antioxidant potential have demonstrated nephroprotective effects in experimental models of diabetes, both of natural [33] and pharmacological [34] origin. This antioxidant potential is of fundamental importance in explaining the nephroprotection of hydroxytyrosol at a morphological and functional level. Moreover, it has been shown that the administration of extra virgin olive oil with a high polyphenol content to patients with chronic kidney disease improves the renal analytical profile to a greater extent than in those patients who were administered extra virgin olive oil with a lower polyphenol content (mainly hydroxytyrosol), directly relating the nephroprotective effect to its antioxidant power [35]. Similarly, our study demonstrates a nephroprotective effect associated with the antioxidant action of hydroxytyrosol, the main polyphenolic compound of virgin olive oil.

Hydroxytyrosol is known to have poor intestinal absorption after oral administration. However, the doses administered in this study must have reached sufficient blood levels since a clear effect is observed on the various biomarkers analyzed.

We must consider two main limitations of this study. First, it has been carried out in an experimental model of type 1 diabetes mellitus, considering that in humans, diabetic nephropathy, due to its higher prevalence, is very common in type 2 diabetes. On the other hand, the time of evolution of diabetes established in the study may have led to less kidney involvement, prolonging the period of diabetes up to 3 months could have been related to more severe kidney involvement.

## 5. Conclusions

Oral administration of 1 and 5 mg/kg/day of hydroxytyrosol produced an antioxidant and nephroprotective effect in an experimental model of type 1-like diabetes mellitus. The nephroprotective effect was significantly associated with the systemic and renal antioxidant action of hydroxytyrosol, as well as its effect on the thromboxane/prostacyclin balance.

## Figures and Tables

**Table 1:** Serum and urine variables (mean ± standard error of the mean) of nondiabetic rats (NDR), diabetic control rats (DR), and DR treated with hydroxytyrosol (HT) 1 mg/kg/day p.o. (HT-1) or 5 mg/kg/day p.o. (HT-5). N = 10 rats per group.

Variable	NDR	DR	*p* vs. NDR	DR + HT-1	*p* vs. DR	DR + HT-5	*p* vs. DR
Serum							
Blood glucose (mg/dL)	90.0 ± 5.5	471 ± 9.9	0.0001	442 ± 30.5	n.s.	451 ± 42.7	n.s.
Creatinine (mg/dL)	0.3 ± 0.01	0.7 ± 0.03	0.0001	0.4 ± 0.04	0.001	0.5 ± 0.04	0.0001
Protein (g/dL)	5.7 ± 0.07	5.5 ± 0.1	n.s.	5.2 ± 0.05	n.s.	5.6 ± 0.2	n.s.
Albumin (g/dL)	1.5 ± 0.08	1.4 ± 0.1	n.s.	1.4 ± 0.08	n.s.	1.5 ± 0.1	n.s.
Urine							
Creatinine (mg/dL)	103 ± 3.7	60.6 ± 3.2	0.0001	72.5 ± 3.1	0.01	74.7 ± 3.8	0.001
Proteinuria (mg/L)	13.1 ± 0.8	91.9 ± 4.7	0.0001	57.8 ± 5.8	0.004	37.7 ± 3.4	0.0001
Proteinuria (mg/24 h)	31.1 ± 8.1	185 ± 17.5	0.005	59.4 ± 7.0	0.004	50.0 ± 2.65	0.008
Glucosuria (mg/L)	0.0 ± 0.0	4065 ± 1611	0.0001	1958 ± 643	n.s.	4752 ± 1803	n.s.
pH	7.8 ± 0.6	7.3 ± 0.8	n.s.	6.9 ± 1.1	n.s.	7.5 ± 0.9	n.s.
8-isoprostane (ng/mg creatinine)	6.9 ± 0.6	49.1 ± 0.6	0.0001	5.2 ± 0.5	0.0001	5.5 ± 0.5	0.0001
11-dH-TxB_2_ (ng/mg creatinine)	4.1 ± 0.8	9.8 ± 0.6	0.003	6.4 ± 1.0	0.045	4.3 ± 0.8	0.009
6-keto-PGF_1__α_ (pg/mg creatinine)	13.8 ± 2.1	7.0 ± 0.5	0.045	8.4 ± 0.7	n.s.	11.8 ± 1.3	0.01

n.s. = no significant differences. 6-keto-PGF_1α_: 6-keto-prostaglandin F_1α;_ 8-isoprostane: 8-iso-prostaglandin F_2α_; 11-dH-TxB_2_: 11-dehydro-tromboxane B_2_.

**Table 2:** Serum and kidney variables (mean ± standard error of the mean) of oxidative and nitrosative stress of nondiabetic rats (NDR), diabetic control rats (DR), and DR treated with hydroxytyrosol (HT) 1 mg/kg/day p.o. (HT-1) or 5 mg/kg/day p.o. (HT-5). N = 10 rats per group.

Variable	NDR	DR	*p* vs. NDR	HT-1	*p* vs. DR	HT-5	*p* vs. DR
Serum							
TBARS (nmol/mL)	4.2 ± 0.4	8.44 ± 0.4	0.0001	6.9 ± 0.8	0.023	4.3 ± 0.3 *	0.0001
oxLDL (ng/mL)	14.6 ± 1.6	24.4 ± 0.7	0.0001	21.5 ± 1.7	n.s.	13.3 ± 0.5 *	0.0001
8-OHdG (ng/mL)	16.1 ± 0.2	26.3 ± 0.8	0.0001	19.6 ± 1.7	0.010	15.4 ± 0.7	0.0001
GHS (nmol/mL)	127 ± 3.9	91.3 ± 3.9	0.0001	109 ± 5.7	0.030	117 ± 7.8	0.02
GSHpx (nmol/min/mL)	7.8 ± 0.6	19.0 ± 1.8	0.0001	11.3 ± 1.3	0.005	11.6 ± 1.7	0.02
TAC (U/mL)	17.9 ± 0.3	13.2 ± 0.4	0.0001	16.6 ± 0.3	0.001	16.6 ± 0.7	0.01
3-nitrotyrosine (pg/mL)	1.5 ± 0.05	6.4 ± 0.3	0.0001	3.2 ± 0.1	0.0001	3.5 ± 0.2	0.0001
Kidney							
TBARS (nmol/mg protein)	35.7 ± 3.4	135 ± 14.2	0.001	61.0 ± 4.4	0.002	44.9 ± 1.8	0.002
8-OHdG (ng/0.1 g tissue)	7.1 ± 0.3	12.6 ± 0.3	0.0001	9.0 ± 0.3	0.0001	8.2 ± 0.3	0.0001
GHS (µmol/0.1 g tissue)	475 ± 12.8	150 ± 10.1	0.0001	289 ± 25.1	0.002	365 ± 20.5	0.0001
GSHpx (nmol/min/0.1 g tissue)	91.4 ± 3.4	65.0 ± 3.1	0.0001	56.4 ± 4.3	n.s.	63.9 ± 3.2	n.s.
TAC (U/0.1 g tissue)	87.2 ± 3.0	40.2 ± 7.6	0.001	58.5 ± 7.0	n.s.	70.1 ± 15.1	n.s.
3-nitrotyrosine (pg/0.1 g tissue)	20.7 ± 0.7	117 ± 6.1	0.0001	81.6 ± 12.0	0.032	41.6 ± 7.6 *	0.001

n.s. = no significant differences. * *p* < 0.05 with respect to HT-1. 8-OHdG: 8-hydroxy-2-deoxyguanosine; GSH: reduced glutathione; GSHpx: glutathione peroxidase activity; oxLDL: oxidized low-density lipoprotein; TAC: total antioxidant capacity; TBARS: thiobarbituric acid reactive substances.

**Table 3:** Pearson correlations between glomerular volume (GV), creatinine clearance (CrCl), and the proteinuria/urine creatinine ratio (Prot/Create) and biochemical variables in serum, kidney, and urine.

Variable	GV		CrCl		Prot/Creat	
	Pc	*p*	Pc	*p*	Pc	*p*
Serum						
TBARS	0.846	0.0001	−0.686	0.005	0.732	0.003
8-HdG	0.888	0.0001	−0.587	0.021	0.764	0.001
oxLDL	0.767	0.0001	−0.560	0.030	0.597	0.024
GSH	−0.829	0.0001	0.639	0.010	−0.810	0.0001
GSHpx	0.820	0.0001	−0.736	0.002	0.786	0.001
TAC	−0.833	0.0001	0.723	0.002	−0.889	0.0001
3-NTy	0.913	0.0001	−0.875	0.0001	0.960	0.0001
Kidney						
TBARS	0.926	0.0001	−0.681	0.005	0.918	0.0001
8-HdG	0.948	0.0001	−0.780	0.001	0.935	0.0001
GSH	−0.953	0.0001	0.816	0.0001	−0.861	0.0001
GSHpx	−0.546	0.035	0.724	0.002	−0.478	0.084
TAC	−0.783	0.001	0.707	0.003	−0.709	0.004
3-NTy	0.844	0.0001	−0.719	0.003	0.769	0.001
Urine						
8-isoprostane	0.856	0.0001	−0.596	0.015	0.859	0.0001
11-dHTxB_2_	0.831	0.0001	−0.602	0.023	0.700	0.005
6-keto-PGF_1α_	−0.636	0.015	0.595	0.025	−0.546	0.043

Pc: Pearson coefficient; *p*: *p* value; TBARS: thiobarbituric acid reactive substances; 8-OHdG: 8-hydroxy-2-oxyguanosine; oxLDL: oxidized low-density lipoprotein; GSH: reduced glutathione; GSHpx: glutathione peroxidase activity; TAC: total antioxidant capacity; 3-Nty: 3-nitrotyrosine; 8-isoprostanes: 8-iso-prostaglandin F_2α;_ 11-dHTxB_2_: 11-dehydro-tromboxane B_2;_ 6-keto-PG_F1α_: 6-keto-prostaglandin F_1._

## Data Availability

Data is contained within the article.
